# Crystal structure of (2-amino-7-methyl-4-oxidopteridine-6-carboxyl­ato-κ^3^ *O* ^4^,*N* ^5^,*O* ^6^)aqua­(1,10-phenanthroline-κ^2^ *N*,*N*′)zinc trihydrate

**DOI:** 10.1107/S2056989015014619

**Published:** 2015-08-12

**Authors:** Siddhartha S. Baisya, Baidyanath Ghosh, Parag S. Roy

**Affiliations:** a Department of Chemistry, University of North Bengal, Siliguri 734 013, India

**Keywords:** crystal structure, phenanthroline, zinc complex, hydrogen bonding, pteridine, π–π stacking

## Abstract

In the title compound, [Zn(C_8_H_5_N_5_O_3_)(C_12_H_8_N_2_)(H_2_O)]·3H_2_O, a tridentate 2-amino-7-methyl-4-oxidopteridine-6-carboxyl­ate ligand, a bidentate ancillary 1,10-phenanthroline (phen) ligand and a water mol­ecule complete a distorted octa­hedral coordination geometry around the Zn^II^ atom. The pterin ligand forms two chelate rings. The phen and pterin ring systems are nearly perpendicular [dihedral angle = 85.16 (5)°]. Classical N—H⋯O, O—H⋯N and O—H⋯O hydrogen bonds and weak C—H⋯O hydrogen bonds link the complex mol­ecules and lattice water mol­ecules into a three-dimensional network. π–π stacking contacts are observed as well, with centroid-to-centroid distances of 3.5679 (14), 3.7004 (14), 3.6641 (15), 3.6974 (13) and 3.3412 (12) Å.

## Related literature   

For the importance of pterin in metalloenzymes, see: Basu & Burgmayer (2011 ▸); Burgmayer (1998 ▸); Fitzpatrick (2003 ▸); Fukuzumi & Kojima (2008 ▸). For the biochemical importance of zinc–pterin inter­actions, see: Chreifi *et al.* (2014 ▸). For the structure of a related zinc complex, see: Mitsumi *et al.* (1995 ▸). For the electron-shuffling ability of the pterin unit, as well as its donor groups, and the effect on the geometric parameters of related complexes, see: Baisya & Roy (2014 ▸); Beddoes *et al.* (1993 ▸); Kohzuma *et al.* (1988 ▸); Miyazaki *et al.* (2008 ▸); Russell *et al.* (1992 ▸). For the synthesis of the pterin ligand, see: Wittle *et al.* (1947 ▸).
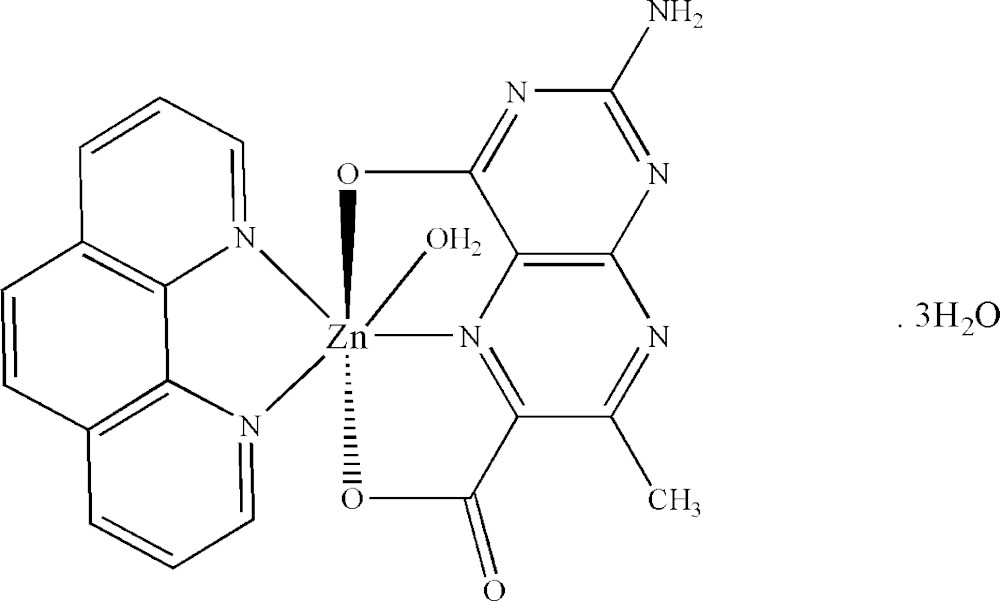


## Experimental   

### Crystal data  

[Zn(C_8_H_5_N_5_O_3_)(C_12_H_8_N_2_)(H_2_O)]·3H_2_O
*M* *_r_* = 536.81
Triclinic, 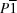

*a* = 8.4819 (7) Å
*b* = 9.9573 (9) Å
*c* = 13.7257 (12) Å
α = 97.667 (1)°
β = 95.243 (1)°
γ = 110.716 (1)°
*V* = 1062.51 (16) Å^3^
*Z* = 2
Mo *K*α radiation
μ = 1.22 mm^−1^
*T* = 293 K
0.24 × 0.19 × 0.04 mm

### Data collection  

Bruker Kappa APEXII diffractometer
Absorption correction: multi-scan (*SADABS*; Bruker, 2001 ▸) *T*_min_ = 0.76, *T*_max_ = 0.95
9149 measured reflections
4794 independent reflections
4456 reflections with *I* > 2.0σ(*I*)
*R*_int_ = 0.018

### Refinement  

*R*[*F* ^2^ > 2σ(*F* ^2^)] = 0.039
*wR*(*F* ^2^) = 0.089
*S* = 0.94
4794 reflections
346 parameters
12 restraints
H atoms treated by a mixture of independent and constrained refinement
Δρ_max_ = 0.64 e Å^−3^
Δρ_min_ = −0.33 e Å^−3^

### 

Data collection: *APEX2* (Bruker, 2007 ▸); cell refinement: *SAINT* (Bruker, 2007 ▸); data reduction: *SAINT*; program(s) used to solve structure: *SHELXS97* (Sheldrick, 2008 ▸); program(s) used to refine structure: *CRYSTALS* (Betteridge *et al.*, 2003 ▸); molecular graphics: *CAMERON* (Watkin *et al.*, 1996 ▸); software used to prepare material for publication: *CRYSTALS*.

## Supplementary Material

Crystal structure: contains datablock(s) global, New_Global_Publ_Block, I. DOI: 10.1107/S2056989015014619/xu5864sup1.cif

Structure factors: contains datablock(s) I. DOI: 10.1107/S2056989015014619/xu5864Isup2.hkl

**Fig. 1.**
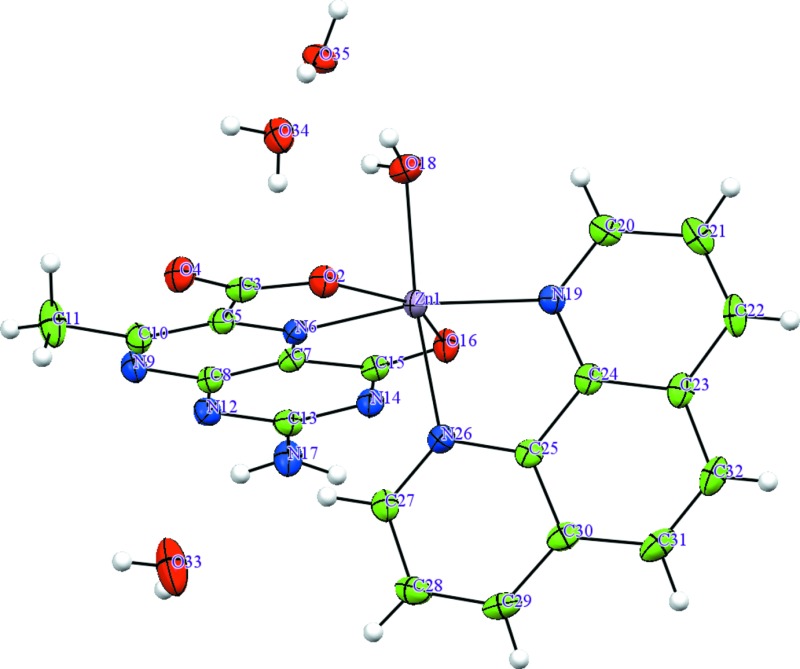
The molecular structure of the title compound. Displacement ellipsoids are drawn at the 50% probability level. Hydrogen atoms are shown as spheres of arbitrary radius.

**Fig. 2.**
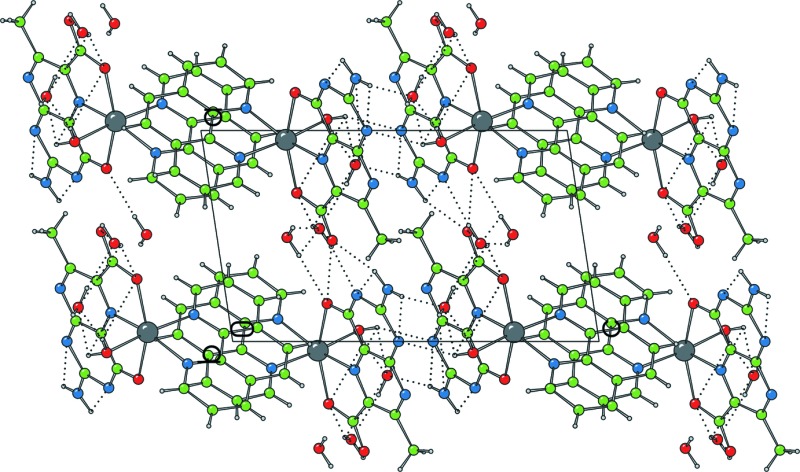
The crystal packing diagram of the title compound, viewed along the b axis. Dotted lines indicate hydrogen bonds.

. DOI: 10.1107/S2056989015014619/xu5864fig1.tif
The mol­ecular structure of the title compound. Displacement ellipsoids are drawn at the 50% probability level. Hydrogen atoms are shown as spheres of arbitrary radius.

b . DOI: 10.1107/S2056989015014619/xu5864fig2.tif
The crystal packing diagram of the title compound, viewed along the *b* axis. Dotted lines indicate hydrogen bonds.

**Fig. 3.**
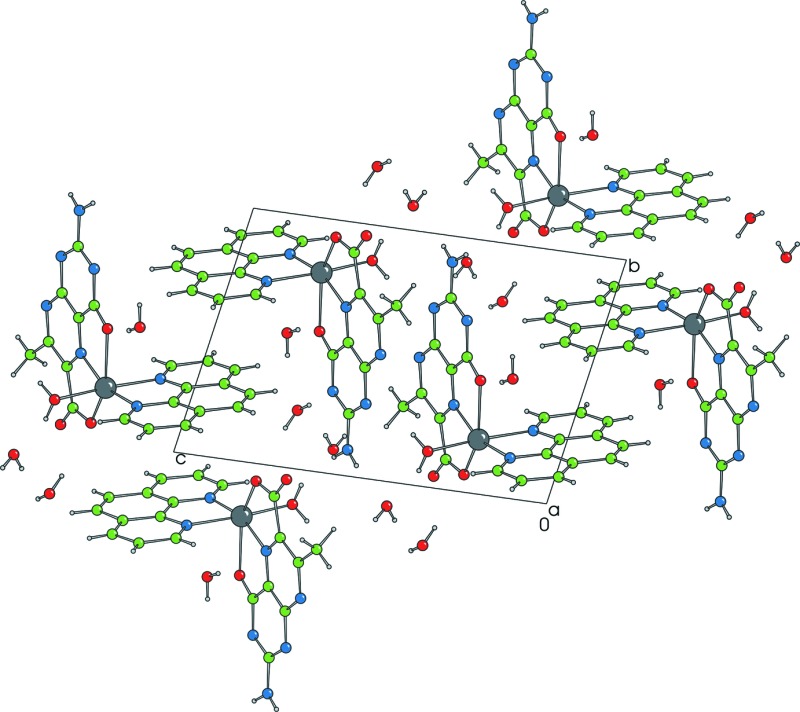
A molecular packing diagram highlighting π–π stacking interactions between two phen–phen and pterin–pterin rings, respectively.

. DOI: 10.1107/S2056989015014619/xu5864fig3.tif
A mol­ecular packing diagram highlighting π–π stacking inter­actions between two phen–phen and pterin–pterin rings, respectively.

CCDC reference: 1416736

Additional supporting information:  crystallographic information; 3D view; checkCIF report

## Figures and Tables

**Table 1:** Selected bond lengths ()

Zn1O2	2.1373(15)
Zn1O16	2.3727(15)
Zn1O18	2.1128(16)
Zn1N6	2.0303(17)
Zn1N19	2.0684(17)
Zn1N26	2.1627(18)

**Table 2:** Hydrogen-bond geometry (, )

*D*H*A*	*D*H	H*A*	*D* *A*	*D*H*A*
N17H171O4^i^	0.85(2)	2.15(2)	2.942(3)	156(2)
N17H172O35^ii^	0.85(3)	2.13(3)	2.967(3)	170(2)
O18H181O35	0.81(2)	1.92(2)	2.700(2)	163(3)
O18H182N12^ii^	0.82(2)	2.30(3)	3.088(2)	160(3)
O33H331O16^iii^	0.81(4)	2.13(4)	2.929(3)	169(4)
O33H332O34^iv^	0.82(3)	2.18(4)	2.944(3)	155(5)
O34H341N14^v^	0.80(3)	2.05(3)	2.842(3)	172(3)
O34H342O2	0.79(3)	2.28(3)	3.010(2)	154(3)
O34H342O4	0.79(3)	2.32(3)	2.950(2)	137(3)
O35H351O34	0.81(3)	1.94(3)	2.735(2)	167(3)
O35H352N12^vi^	0.81(3)	2.06(3)	2.855(3)	168(3)
C20H201O4^vii^	0.93	2.49	3.186(3)	132
C27H271O33	0.92	2.56	3.360(4)	146
C29H291O16^viii^	0.91	2.55	3.394(3)	156

Symmetry codes: (i) 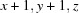; (ii) 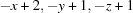; (iii) 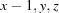; (iv) 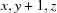; (v) 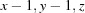; (vi) 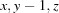; (vii) 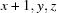; (viii) 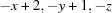.

## supplementary crystallographic information

### S1. Comment

Pterins (2- amino -4- oxidopteridines) are present in a wide range of biological functions including a large number of metalloenzymes (Basu & Burgmayer, 2011; Burgmayer, 1998; Fitzpatrick, 2003; Fukuzumi & Kojima, 2008). Even the biochemical importance of zinc–pterin inter­action has been established X-ray structurally (Chreifi *et al.*, 2014). Literature survey revels the existence of only one x-ray structurally characterized zinc(II)–pterin complex (Mitsumi *et al.*, 1995). The present effort is concerned with the title complex, possessing both a tridentate pterin ligand and a π-acceptor ancillary ligand like 1, 10—phenanthroline (phen). The six-coordinated Zn^II^ atom exhibits departure from a regular o­cta­hedral geometry with respect to both bond lengths and angles (Fig. 1). The equatorial plane is formed by the two N atoms (N19, N26) of phen, the pyrazine ring N atom (N6) of the pterin ligand and the aqua O atom (O18). The axial positions are occupied by the two pterin O atoms (O2 and O16), with the latter one forming the longest axial bond [2.3724 (16) Å]. One important reason causing distortion from regular o­cta­hedral geometry is that this pterin ligand forms two five-membered chelate rings with small bite angles [76.28 (7) and 74.66 (6)°], instead of only one per pterin ligand for the earlier case (Mitsumi *et al.*, 1995). A consideration of the charge balance of this complex indicates that this pterin ligand acts as a binegative tridentate ONO-donor. A near orthogonal disposition of the phen ligand and pterin chelate ring is observed, which affords minimum steric repulsion. Of the three axes, least deviation from linearity is observed in the O18—Zn1—N26 direction [173.36 (7)°].

The exocyclic bond length data of the pyrimidine ring, C15—O16 [1.257 (3) Å] and C13—N17 [1.335 (3) Å] merit attention. Participation by the pterin unit in the electron-shuffling process from the pyrazin ring N9 to the C15 carbonyl group is indicated, as suggested in the literature (Baisya & Roy, 2014; Beddoes *et al.*, 1993; Kohzuma *et al.*, 1988; Miyazaki *et al.*, 2008; Russell *et al.*, 1992). Formation of the Zn1—O16 bond assists this process.

In the crystal, the complex molecules and lattice water molecules are linked by inter­molecular N—H ··· O, O—H ··· N and O—H ··· O hydrogen bonds (Table 1) into a three-dimensional network. The lattice water molecules play a decisive role in the crystal packing process (Fig. 2). Fig. 3 indicates π-π stacking inter­actions involving two parallel, inversion-related pterin rings within the same unit cell and showing face-to-face distance of 3.6974 (13) and 3.3412 (12) Å. Besides this, the nearly parallel phen rings of adjacent molecules also display π–π stacking inter­actions with centroid–centroid distance of 3.5678 (14), 3.7004 (14) and 3.6641 (15) Å.

### S2. Experimental

2-Amino-4-hy­droxy-7-methyl­pteridine-6-carb­oxy­lic acid sesquihydrate (C_8_H_7_N_5_O_3_.1.5H_2_O) was obtained by published procedure (Wittle *et al.* 1947). The title complex was prepared by the dropwise addition of a solution (15 ml) of ZnSO_4_. 7H_2_O (35.9 mg, 0.125 mmol) containing 1,10-phenanthroline monohydrate (25 mg, 0.125 mmol) to a warm (312 K) aqueous alkaline solution (NaOH: 11 mg, 0.275 mmol) of the pterin ligand (31 mg, 0.125 mmol). The pH value was maintained around 9.9–10.0 and the final volume was 60 ml. The reaction mixture was transferred to a 100 ml beaker and allowed to stand at room temperature. Light brown shining crystals suitable for single crystal X-ray diffraction appeared after 10 days (yield 30%).

### S2.1. Refinement

The H atoms were all located in a difference map, but those attached to carbon atoms were repositioned geometrically. The H atoms were initially refined with soft restraints on the bond lengths and angles to regularise their geometry (C—H in the range 0.93–0.98 Å, N—H in the range 0.86–0.89 Å and O—H = 0.82 Å) and *U*_iso_(H) (in the range 1.2–1.5 times *U*_eq_ of the parent atom), after which the positions were refined with riding constraints.

### Crystal data

**Table:**

[Zn(C_8_H_5_N_5_O_3_)(C_12_H_8_N_2_(H_2_O)]·3H_2_O	*Z* = 2
*M**_r_* = 536.81	*F*(000) = 552
Triclinic, *P*1	*D*_x_ = 1.678 Mg m^−^^3^
Hall symbol: -P 1	Mo *K*α radiation, λ = 0.71073 Å
*a* = 8.4819 (7) Å	Cell parameters from 0 reflections
*b* = 9.9573 (9) Å	θ = 0–0°
*c* = 13.7257 (12) Å	µ = 1.22 mm^−^^1^
α = 97.667 (1)°	*T* = 293 K
β = 95.243 (1)°	Plate, orange brown
γ = 110.716 (1)°	0.24 × 0.19 × 0.04 mm
*V* = 1062.51 (16) Å^3^	

### Data collection

**Table:**

Bruker Kappa APEXII diffractometer	4456 reflections with *I* > 2.0σ(*I*)
Graphite monochromator	*R*_int_ = 0.018
φ & ω scans	θ_max_ = 28.3°, θ_min_ = 1.5°
Absorption correction: multi-scan (*SADABS*; Bruker, 2001)	*h* = −11→11
*T*_min_ = 0.76, *T*_max_ = 0.95	*k* = −13→13
9149 measured reflections	*l* = −17→18
4794 independent reflections	

### Refinement

**Table:**

Refinement on *F*^2^	Primary atom site location: structure-invariant direct methods
Least-squares matrix: full	Hydrogen site location: difference Fourier map
*R*[*F*^2^ > 2σ(*F*^2^)] = 0.039	H atoms treated by a mixture of independent and constrained refinement
*wR*(*F*^2^) = 0.089	Method, part 1, Chebychev polynomial, (Watkin, 1994, *P*rince, 1982) [*w*eight] = 1.0/[A_0_*T_0_(x) + A_1_*T_1_(x) ··· + A_n-1_]*T_n-1_(x)] where A_i_ are the Chebychev coefficients listed belo*w* and x = *F* /*F*max Method = Robust Weighting (*P*rince, 1982) W = [*w*eight] * [1-(delta*F*/6*sigma*F*)^2^]^2^ A_i_ are: 57.7 96.4 59.4 25.6 6.16
*S* = 0.94	(Δ/σ)_max_ = 0.001
4794 reflections	Δρ_max_ = 0.64 e Å^−^^3^
346 parameters	Δρ_min_ = −0.33 e Å^−^^3^
12 restraints	

### Fractional atomic coordinates and isotropic or equivalent isotropic displacement parameters (Å^2^)

**Table:**

	*x*	*y*	*z*	*U*_iso_*/*U*_eq_	
Zn1	0.95623 (3)	0.21520 (3)	0.228578 (17)	0.0166	
O2	0.70435 (19)	0.06939 (16)	0.23606 (11)	0.0206	
C3	0.6217 (3)	0.1186 (2)	0.29541 (15)	0.0182	
O4	0.47875 (19)	0.04655 (17)	0.31282 (12)	0.0231	
C5	0.7109 (3)	0.2776 (2)	0.34528 (15)	0.0164	
N6	0.8648 (2)	0.33756 (18)	0.32071 (12)	0.0149	
C7	0.9604 (3)	0.4752 (2)	0.35603 (14)	0.0155	
C8	0.9030 (3)	0.5640 (2)	0.41988 (15)	0.0169	
N9	0.7480 (2)	0.5050 (2)	0.44879 (14)	0.0198	
C10	0.6531 (3)	0.3650 (2)	0.41309 (16)	0.0191	
C11	0.4848 (3)	0.3041 (3)	0.4490 (2)	0.0308	
H111	0.3927	0.2783	0.3982	0.0483*	
H113	0.4752	0.3721	0.4998	0.0481*	
H112	0.4768	0.2178	0.4756	0.0485*	
N12	0.9998 (2)	0.70678 (19)	0.45260 (13)	0.0182	
C13	1.1515 (3)	0.7547 (2)	0.41752 (15)	0.0180	
N14	1.2199 (2)	0.67528 (19)	0.35785 (13)	0.0175	
C15	1.1294 (3)	0.5323 (2)	0.32689 (15)	0.0168	
O16	1.17955 (19)	0.44769 (16)	0.27321 (11)	0.0198	
N17	1.2464 (2)	0.8967 (2)	0.44431 (15)	0.0227	
H172	1.213 (3)	0.954 (2)	0.481 (2)	0.0276*	
H171	1.334 (3)	0.933 (2)	0.4174 (19)	0.0277*	
O18	1.0532 (2)	0.14070 (18)	0.34889 (12)	0.0218	
H181	0.989 (4)	0.061 (2)	0.355 (2)	0.0355*	
H182	1.063 (4)	0.196 (3)	0.4008 (17)	0.0360*	
N19	1.1061 (2)	0.15973 (19)	0.13227 (13)	0.0168	
C20	1.2233 (3)	0.1030 (2)	0.15288 (16)	0.0204	
C21	1.3255 (3)	0.0777 (3)	0.08353 (18)	0.0250	
C22	1.3061 (3)	0.1119 (3)	−0.00880 (17)	0.0250	
C23	1.1848 (3)	0.1743 (2)	−0.03259 (16)	0.0200	
C24	1.0876 (3)	0.1965 (2)	0.04125 (15)	0.0171	
C25	0.9617 (3)	0.2597 (2)	0.02089 (15)	0.0165	
N26	0.8729 (2)	0.27983 (19)	0.09497 (13)	0.0174	
C27	0.7551 (3)	0.3368 (2)	0.07879 (16)	0.0211	
C28	0.7189 (3)	0.3760 (3)	−0.01264 (18)	0.0260	
C29	0.8072 (3)	0.3552 (2)	−0.08843 (17)	0.0247	
C30	0.9340 (3)	0.2960 (2)	−0.07305 (16)	0.0210	
C31	1.0338 (3)	0.2694 (3)	−0.14734 (17)	0.0280	
C32	1.1539 (3)	0.2124 (3)	−0.12796 (17)	0.0266	
H321	1.2157	0.1968	−0.1756	0.0332*	
H311	1.0138	0.2929	−0.2093	0.0332*	
H291	0.7852	0.3813	−0.1476	0.0296*	
H281	0.6360	0.4140	−0.0213	0.0316*	
H271	0.6959	0.3519	0.1296	0.0262*	
H221	1.3708	0.0958	−0.0549	0.0310*	
H211	1.4043	0.0380	0.1006	0.0321*	
H201	1.2367	0.0788	0.2153	0.0258*	
O33	0.4935 (3)	0.4716 (2)	0.1913 (2)	0.0499	
H331	0.407 (4)	0.454 (5)	0.216 (3)	0.0756*	
H332	0.535 (6)	0.560 (2)	0.211 (4)	0.0757*	
O34	0.5353 (2)	−0.23088 (17)	0.28297 (13)	0.0244	
H341	0.452 (3)	−0.257 (4)	0.309 (2)	0.0388*	
H342	0.547 (4)	−0.156 (3)	0.265 (2)	0.0388*	
O35	0.8383 (2)	−0.09668 (17)	0.40775 (12)	0.0225	
H351	0.747 (3)	−0.148 (3)	0.375 (2)	0.0346*	
H352	0.874 (4)	−0.156 (3)	0.426 (2)	0.0349*	

### Atomic displacement parameters (Å^2^)

**Table:**

	*U*^11^	*U*^22^	*U*^33^	*U*^12^	*U*^13^	*U*^23^
Zn1	0.01928 (13)	0.01775 (13)	0.01479 (12)	0.00892 (9)	0.00487 (8)	0.00239 (8)
O2	0.0205 (7)	0.0176 (7)	0.0217 (7)	0.0054 (6)	0.0039 (6)	0.0014 (6)
C3	0.0189 (10)	0.0180 (10)	0.0177 (9)	0.0069 (8)	−0.0001 (7)	0.0047 (8)
O4	0.0172 (7)	0.0204 (8)	0.0276 (8)	0.0015 (6)	0.0054 (6)	0.0043 (6)
C5	0.0157 (9)	0.0164 (9)	0.0174 (9)	0.0058 (7)	0.0024 (7)	0.0045 (7)
N6	0.0156 (8)	0.0158 (8)	0.0138 (7)	0.0060 (6)	0.0031 (6)	0.0033 (6)
C7	0.0172 (9)	0.0174 (9)	0.0122 (8)	0.0062 (7)	0.0025 (7)	0.0036 (7)
C8	0.0184 (9)	0.0176 (9)	0.0164 (9)	0.0082 (8)	0.0031 (7)	0.0039 (7)
N9	0.0184 (8)	0.0188 (8)	0.0239 (9)	0.0083 (7)	0.0070 (7)	0.0031 (7)
C10	0.0163 (9)	0.0209 (10)	0.0218 (10)	0.0078 (8)	0.0061 (8)	0.0043 (8)
C11	0.0196 (11)	0.0247 (11)	0.0435 (14)	0.0035 (9)	0.0146 (10)	−0.0026 (10)
N12	0.0199 (8)	0.0163 (8)	0.0188 (8)	0.0073 (7)	0.0040 (7)	0.0023 (7)
C13	0.0191 (9)	0.0185 (10)	0.0172 (9)	0.0079 (8)	0.0015 (7)	0.0041 (8)
N14	0.0180 (8)	0.0149 (8)	0.0188 (8)	0.0050 (7)	0.0048 (6)	0.0023 (6)
C15	0.0182 (9)	0.0177 (9)	0.0158 (9)	0.0071 (7)	0.0037 (7)	0.0057 (7)
O16	0.0197 (7)	0.0164 (7)	0.0229 (7)	0.0057 (6)	0.0088 (6)	0.0011 (6)
N17	0.0220 (9)	0.0152 (8)	0.0283 (10)	0.0036 (7)	0.0091 (8)	0.0000 (7)
O18	0.0228 (8)	0.0211 (8)	0.0204 (7)	0.0060 (6)	0.0029 (6)	0.0065 (6)
N19	0.0191 (8)	0.0142 (8)	0.0159 (8)	0.0062 (6)	0.0015 (6)	−0.0002 (6)
C20	0.0201 (10)	0.0187 (10)	0.0205 (10)	0.0071 (8)	−0.0013 (8)	−0.0001 (8)
C21	0.0200 (10)	0.0238 (11)	0.0308 (12)	0.0107 (9)	0.0013 (9)	−0.0026 (9)
C22	0.0207 (10)	0.0257 (11)	0.0259 (11)	0.0081 (9)	0.0079 (8)	−0.0065 (9)
C23	0.0189 (10)	0.0184 (9)	0.0187 (9)	0.0035 (8)	0.0047 (8)	−0.0012 (8)
C24	0.0170 (9)	0.0153 (9)	0.0155 (9)	0.0034 (7)	0.0017 (7)	−0.0010 (7)
C25	0.0162 (9)	0.0139 (9)	0.0173 (9)	0.0035 (7)	0.0025 (7)	0.0015 (7)
N26	0.0178 (8)	0.0183 (8)	0.0160 (8)	0.0063 (7)	0.0031 (6)	0.0032 (6)
C27	0.0190 (10)	0.0228 (10)	0.0230 (10)	0.0090 (8)	0.0055 (8)	0.0040 (8)
C28	0.0233 (11)	0.0261 (11)	0.0307 (12)	0.0107 (9)	0.0004 (9)	0.0100 (9)
C29	0.0262 (11)	0.0241 (11)	0.0216 (10)	0.0060 (9)	−0.0016 (9)	0.0099 (9)
C30	0.0225 (10)	0.0178 (10)	0.0188 (10)	0.0025 (8)	0.0015 (8)	0.0047 (8)
C31	0.0355 (13)	0.0291 (12)	0.0161 (10)	0.0069 (10)	0.0050 (9)	0.0058 (9)
C32	0.0296 (12)	0.0292 (12)	0.0187 (10)	0.0076 (9)	0.0098 (9)	0.0017 (9)
O33	0.0397 (12)	0.0342 (11)	0.0765 (16)	0.0141 (9)	0.0294 (11)	−0.0026 (11)
O34	0.0200 (8)	0.0188 (8)	0.0328 (9)	0.0042 (6)	0.0082 (6)	0.0043 (7)
O35	0.0222 (8)	0.0172 (7)	0.0278 (8)	0.0072 (6)	0.0017 (6)	0.0048 (6)

### Geometric parameters (Å, º)

**Table:**

Zn1—O2	2.1373 (15)	N19—C20	1.332 (3)
Zn1—O16	2.3727 (15)	N19—C24	1.359 (3)
Zn1—O18	2.1128 (16)	C20—C21	1.401 (3)
Zn1—N6	2.0303 (17)	C20—H201	0.928
Zn1—N19	2.0684 (17)	C21—C22	1.366 (4)
Zn1—N26	2.1627 (18)	C21—H211	0.917
O2—C3	1.279 (3)	C22—C23	1.413 (3)
C3—O4	1.235 (3)	C22—H221	0.909
C3—C5	1.522 (3)	C23—C24	1.406 (3)
C5—N6	1.325 (3)	C23—C32	1.437 (3)
C5—C10	1.426 (3)	C24—C25	1.442 (3)
N6—C7	1.317 (3)	C25—N26	1.355 (3)
C7—C8	1.401 (3)	C25—C30	1.406 (3)
C7—C15	1.459 (3)	N26—C27	1.327 (3)
C8—N9	1.357 (3)	C27—C28	1.402 (3)
C8—N12	1.354 (3)	C27—H271	0.923
N9—C10	1.335 (3)	C28—C29	1.372 (3)
C10—C11	1.499 (3)	C28—H281	0.916
C11—H111	0.934	C29—C30	1.410 (3)
C11—H113	0.936	C29—H291	0.909
C11—H112	0.960	C30—C31	1.439 (3)
N12—C13	1.362 (3)	C31—C32	1.353 (4)
C13—N14	1.368 (3)	C31—H311	0.929
C13—N17	1.335 (3)	C32—H321	0.906
N14—C15	1.342 (3)	O33—H331	0.803 (19)
C15—O16	1.257 (3)	O33—H332	0.816 (19)
N17—H172	0.851 (17)	O34—H341	0.800 (18)
N17—H171	0.848 (17)	O34—H342	0.795 (18)
O18—H181	0.809 (17)	O35—H351	0.813 (17)
O18—H182	0.817 (17)	O35—H352	0.803 (17)
			
O2—Zn1—N6	76.37 (6)	C13—N17—H171	119.4 (14)
O2—Zn1—O16	150.97 (6)	H172—N17—H171	119 (2)
N6—Zn1—O16	74.60 (6)	Zn1—O18—H181	112 (2)
O2—Zn1—O18	90.05 (6)	Zn1—O18—H182	110 (2)
N6—Zn1—O18	91.77 (7)	H181—O18—H182	106 (3)
O16—Zn1—O18	91.21 (6)	Zn1—N19—C20	127.00 (15)
O2—Zn1—N19	121.85 (6)	Zn1—N19—C24	114.19 (14)
N6—Zn1—N19	160.68 (7)	C20—N19—C24	118.63 (18)
O16—Zn1—N19	86.96 (6)	N19—C20—C21	122.3 (2)
O18—Zn1—N19	94.39 (7)	N19—C20—H201	118.6
O2—Zn1—N26	92.53 (6)	C21—C20—H201	119.1
N6—Zn1—N26	94.76 (7)	C20—C21—C22	119.6 (2)
O16—Zn1—N26	89.49 (6)	C20—C21—H211	119.5
O18—Zn1—N26	173.38 (6)	C22—C21—H211	120.8
N19—Zn1—N26	79.07 (7)	C21—C22—C23	119.5 (2)
Zn1—O2—C3	115.97 (13)	C21—C22—H221	120.8
O2—C3—O4	124.45 (19)	C23—C22—H221	119.6
O2—C3—C5	115.38 (18)	C22—C23—C24	117.3 (2)
O4—C3—C5	120.17 (19)	C22—C23—C32	123.6 (2)
C3—C5—N6	112.43 (17)	C24—C23—C32	119.1 (2)
C3—C5—C10	129.33 (19)	C23—C24—N19	122.57 (19)
N6—C5—C10	118.23 (18)	C23—C24—C25	119.65 (19)
C5—N6—Zn1	119.65 (14)	N19—C24—C25	117.78 (18)
C5—N6—C7	120.98 (18)	C24—C25—N26	117.17 (18)
Zn1—N6—C7	119.37 (14)	C24—C25—C30	120.11 (19)
N6—C7—C8	121.49 (19)	N26—C25—C30	122.71 (19)
N6—C7—C15	117.67 (18)	Zn1—N26—C25	111.62 (13)
C8—C7—C15	120.84 (18)	Zn1—N26—C27	129.44 (14)
C7—C8—N9	119.03 (19)	C25—N26—C27	118.92 (18)
C7—C8—N12	120.91 (19)	N26—C27—C28	122.2 (2)
N9—C8—N12	120.06 (18)	N26—C27—H271	118.9
C8—N9—C10	118.80 (18)	C28—C27—H271	118.9
C5—C10—N9	121.40 (19)	C27—C28—C29	119.4 (2)
C5—C10—C11	121.82 (19)	C27—C28—H281	119.8
N9—C10—C11	116.77 (19)	C29—C28—H281	120.8
C10—C11—H111	112.4	C28—C29—C30	119.6 (2)
C10—C11—H113	110.0	C28—C29—H291	119.8
H111—C11—H113	109.1	C30—C29—H291	120.6
C10—C11—H112	109.7	C29—C30—C25	117.2 (2)
H111—C11—H112	107.5	C29—C30—C31	124.2 (2)
H113—C11—H112	107.9	C25—C30—C31	118.7 (2)
C8—N12—C13	114.98 (17)	C30—C31—C32	121.4 (2)
N12—C13—N14	128.05 (19)	C30—C31—H311	118.0
N12—C13—N17	116.57 (19)	C32—C31—H311	120.6
N14—C13—N17	115.38 (19)	C23—C32—C31	121.1 (2)
C13—N14—C15	118.29 (18)	C23—C32—H321	118.9
C7—C15—N14	116.72 (18)	C31—C32—H321	120.1
C7—C15—O16	119.05 (18)	H331—O33—H332	99 (5)
N14—C15—O16	124.20 (19)	H341—O34—H342	110 (3)
Zn1—O16—C15	109.18 (13)	H351—O35—H352	102 (3)
C13—N17—H172	121.5 (14)		

### Hydrogen-bond geometry (Å, º)

**Table:**

*D*—H···*A*	*D*—H	H···*A*	*D*···*A*	*D*—H···*A*
N17—H171···O4^i^	0.85 (2)	2.15 (2)	2.942 (3)	156 (2)
N17—H172···O35^ii^	0.85 (3)	2.13 (3)	2.967 (3)	170 (2)
O18—H181···O35	0.81 (2)	1.92 (2)	2.700 (2)	163 (3)
O18—H182···N12^ii^	0.82 (2)	2.30 (3)	3.088 (2)	160 (3)
O33—H331···O16^iii^	0.81 (4)	2.13 (4)	2.929 (3)	169 (4)
O33—H332···O34^iv^	0.82 (3)	2.18 (4)	2.944 (3)	155 (5)
O34—H341···N14^v^	0.80 (3)	2.05 (3)	2.842 (3)	172 (3)
O34—H342···O2	0.79 (3)	2.28 (3)	3.010 (2)	154 (3)
O34—H342···O4	0.79 (3)	2.32 (3)	2.950 (2)	137 (3)
O35—H351···O34	0.81 (3)	1.94 (3)	2.735 (2)	167 (3)
O35—H352···N12^vi^	0.81 (3)	2.06 (3)	2.855 (3)	168 (3)
C20—H201···O4^vii^	0.93	2.49	3.186 (3)	132
C27—H271···O33	0.92	2.56	3.360 (4)	146
C29—H291···O16^viii^	0.91	2.55	3.394 (3)	156

Symmetry codes: (i) *x*+1, *y*+1, *z*; (ii) −*x*+2, −*y*+1, −*z*+1; (iii) *x*−1, *y*, *z*; (iv) *x*, *y*+1, *z*; (v) *x*−1, *y*−1, *z*; (vi) *x*, *y*−1, *z*; (vii) *x*+1, *y*, *z*; (viii) −*x*+2, −*y*+1, −*z*.
