# Supplementary figures and images for: Crystal structure of (2-amino-7-methyl-4-oxidopteridine-6-carboxyl­ato-κ3 O 4,N 5,O 6)aqua­(1,10-phenanthroline-κ2 N,N′)zinc trihydrate
Source: Acta Crystallogr E Crystallogr Commun. 2015 Aug 12;71(Pt 9):m162–3. doi: 10.1107/S2056989015014619 (PMC4555367; doi:10.1107/S2056989015014619)

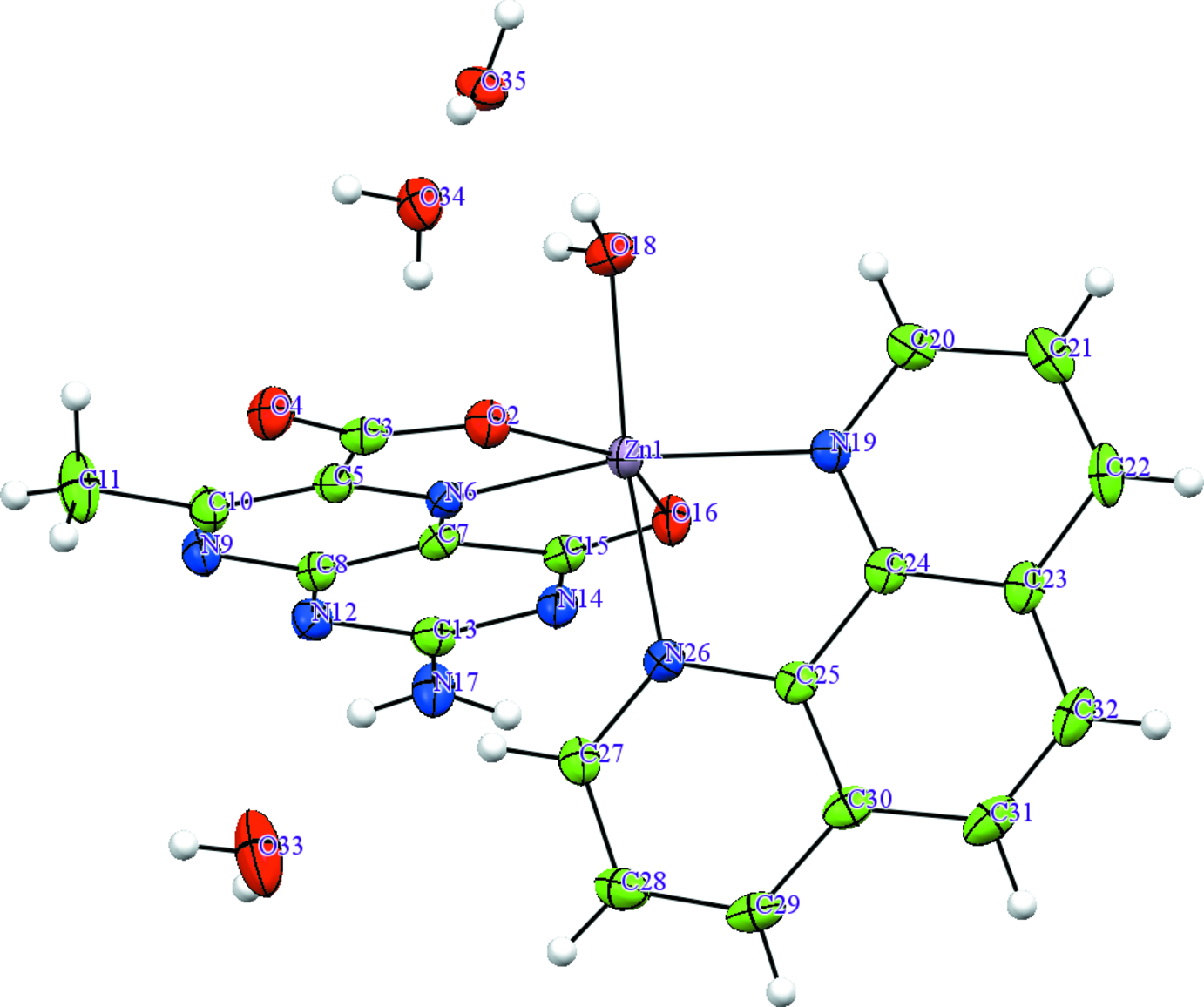

Supplement: Supplementary file 3 [file e-71-0m162-fig1.tif]

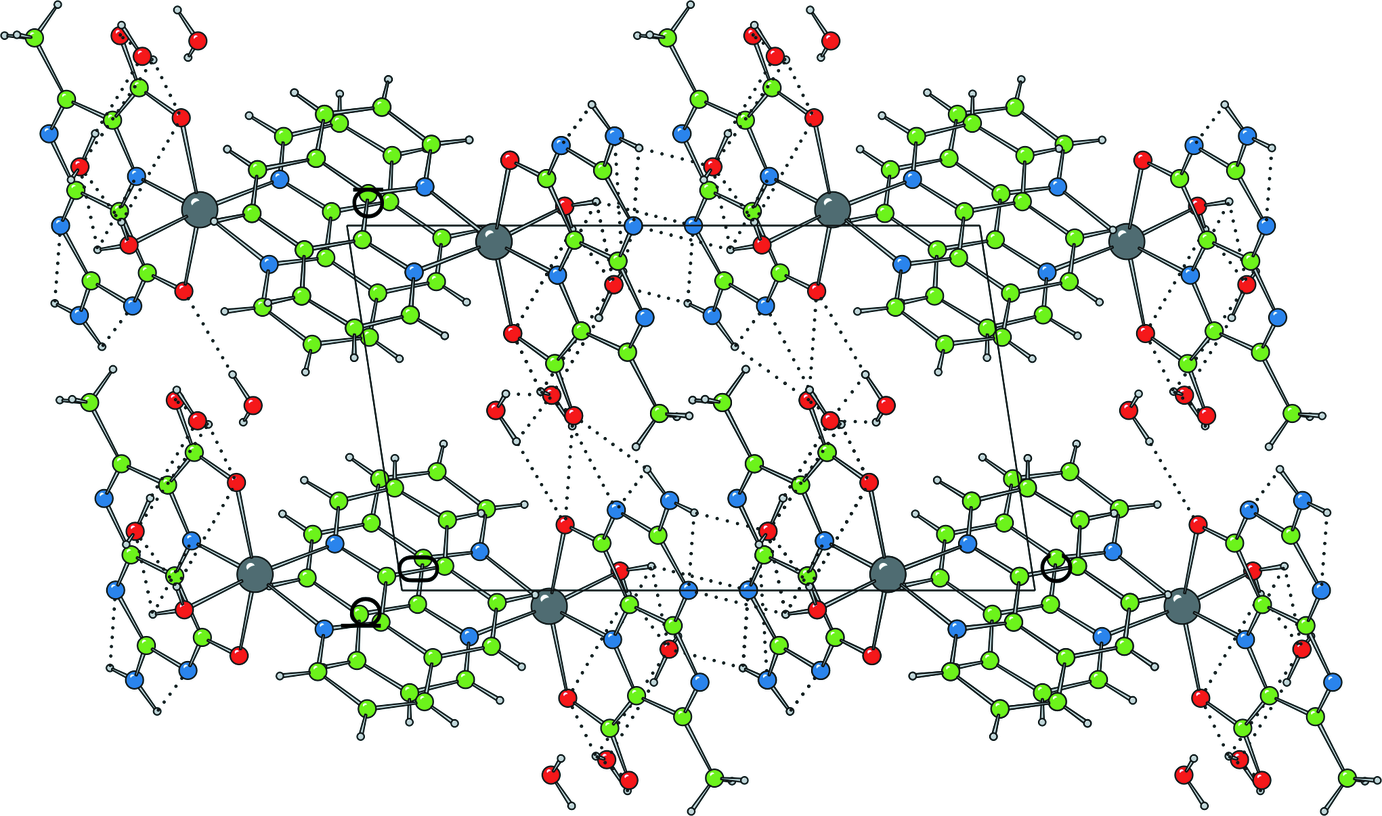

Supplement: Supplementary file 4 [file e-71-0m162-fig2.tif]

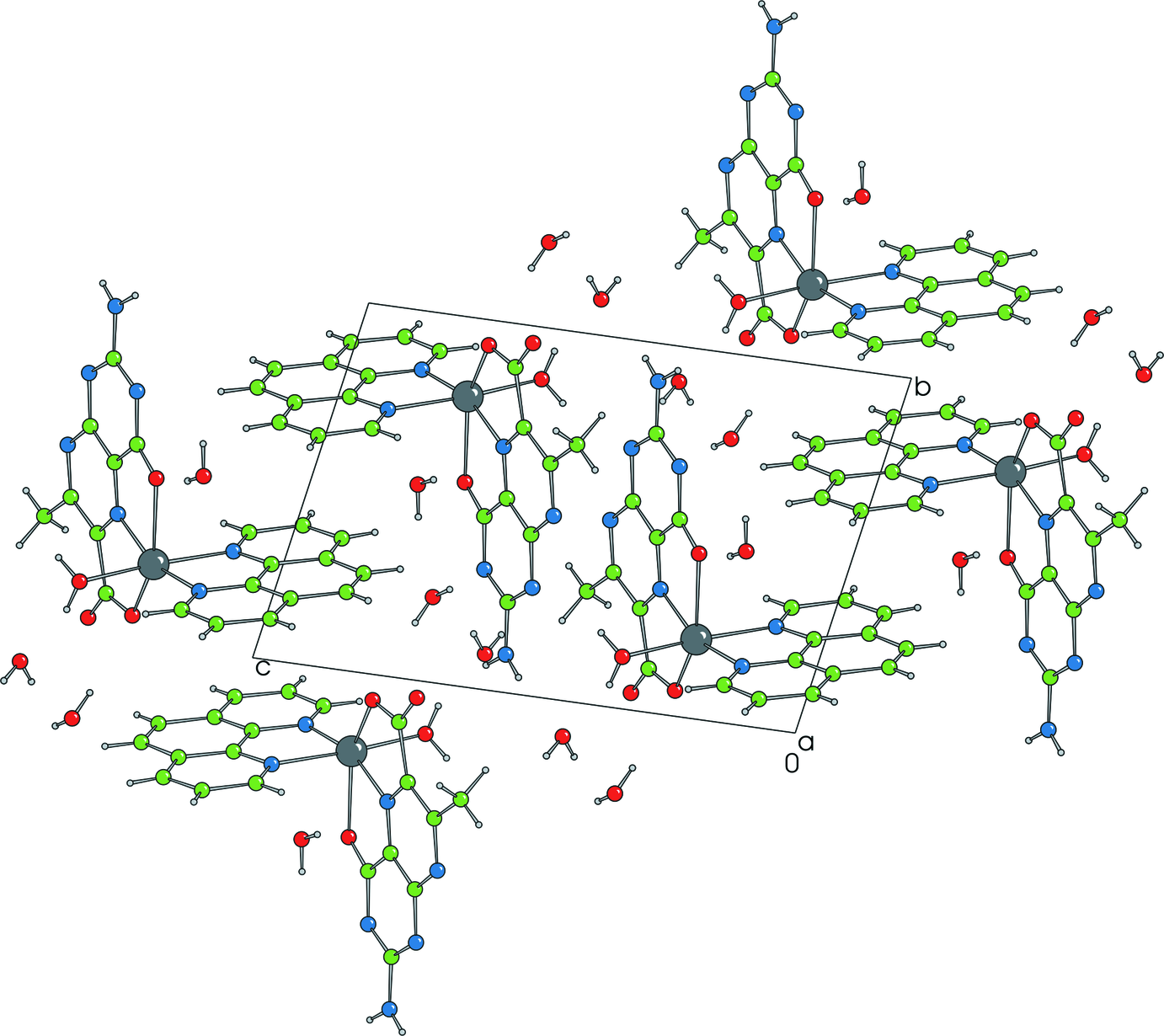

Supplement: Supplementary file 5 [file e-71-0m162-fig3.tif]
